# Dental Students in Baltimore City

**Published:** 1883-12

**Authors:** 


### EDITORIAL, ETC.

DENTAL STUDENTS IN BALTIMORE CITY.—More Dental Students are in this city at the different dental schools than ever before, the majority being at the University of Maryland Dental Department, where there are now over *eighty matriculates* for present session, 1883–84, quite a large increase over last year. All the matriculates at the University of Maryland Dental Department are *purely* dental students.

The organization of the Dental Department of the University has had the effect of bringing more dental students to this city than were ever here before during a college session.

The success which has attended this University Dental Department is indeed unprecedented. What is very encouraging is the fact that almost the entire number of members of the junior class of last session, are present this ession as members of the senior class.

